# Prevalence of *Salmonella* in poultry processing environments in wet markets in Penang and Perlis, Malaysia

**DOI:** 10.14202/vetworld.2017.286-292

**Published:** 2017-03-06

**Authors:** Hafiz Nidaullah, Nadarajan Abirami, Ahamed Kamal Shamila-Syuhada, Li-Oon Chuah, Huda Nurul, Teik Pei Tan, Farah Wahida Zainal Abidin, Gulam Rusul

**Affiliations:** 1 Food Technology Division, School of Industrial Technology, Universiti Sains Malaysia, 11800 Minden, Penang, Malaysia; 2 Public Health Laboratory, Ministry of Health, Jalan Jelapang, 30020 Ipoh, Perak, Malaysia

**Keywords:** prevalence, poultry, *Salmonella*, wet markets

## Abstract

**Aim:**

The aim of this study was to determine the prevalence of various *Salmonella* serotypes in chickens, carcass contact surfaces as well as environmental samples collected from wet markets and small scale processing plant.

**Materials and Methods:**

A total of 182 poultry and environmental samples were collected at random on separate occasions from wet markets and small scale processing plant, during the period of October 2014 to July 2015 in Penang and Perlis, Malaysia. The samples were analyzed for the presence of *Salmonella* using ISO 6579:2002 conventional culture-based method. Presumptive *Salmonella* colonies were subjected to various biochemical tests (such as triple sugar iron and lysine iron test), serologically confirmed using polyvalent O and H antisera and further serotyped at Public Health Laboratory, Ministry of Health, Perak, Malaysia.

**Results:**

*Salmonella* serotypes were isolated from 161 out of 182 samples (88.46%) with 100% prevalence in the whole chicken carcass and chicken cuts - as well as transport crate, cage, drum, knife, chopping board, display table, floor, bench wash water, wash water, and drain water. *Salmonella* was isolated from 91.67%, 83.33%, and 66.67% of defeathering machines, drain swabs, and apron, respectively. 17 serotypes were isolated in this study with *Salmonella* Albany (57/161), *Salmonella* Corvallis (42/161), and *Salmonella* Brancaster (37/161) being the predominant serovars.

**Conclusion:**

The most carcass contact and environmental samples collected along the wet market chicken processing line were consistently contaminated with *Salmonella*. This indicates that *Salmonella* has established itself in poultry processing environments by colonizing the surfaces of the equipment and survives in these environments by establishing biofilms. Our results highlight the need of implementing strict hygiene and sanitation standards to reduce the incidence of *Salmonella*. The prevalence of *Salmonella* in poultry can be reduced effectively by identifying and eliminating the sources and contamination sites during slaughter and processing of poultry.

## Introduction

*Salmonella* is a leading cause of foodborne illness worldwide with an estimated annual economic loss of 3.7 billion dollars [1]. Although diseases due to this pathogen have been associated with a wide variety of food sources, poultry, in particular, have been regarded as the single largest cause of human salmonellosis [2]. In developing countries, such as Malaysia, poultry and poultry products are cheap and staple source of animal protein for all ethnic groups. Malaysia produces an estimate of 1.44 million tons of poultry meat and the consumption per capita are 40 kg/year, one of the highest in the world [3]. While most poultry meat sold are processed in large-scale poultry processing facilities, wet markets still serve as an essential outlet for poultry distribution in Malaysia, accounting for 40% of total sales [3]. Wet markets are very common and popular in Asian countries. Wet markets serve as an avenue for retailing fresh produce, animal, and sea foods. The products are sold at ambient temperature and exposed to the environment. Ice is rarely used for chilling fresh produce or animal products, except for sea foods which are usually covered by ice. In most wet markets in Malaysia, the slaughter of poultry is not allowed or encouraged by local authorities but as Malaysians prefer freshly slaughtered poultry meat, thus poultry is slaughtered and retail in wet markets. The raw foods retailed at wet markets and floors of wet markets are constantly sprayed with water for washing and to maintain the humidity. As raw foods are exposed to the elements together with poor or inadequate hygienic measures in the local markets, we are of opinion there is a very high risk of *Salmonella* contamination of poultry sold in wet markets and should be of great concern to both the public and regulatory agencies.

The prevalence of various *Salmonella* serotypes among live birds ranges from 6% to 30% [4-7], while the incidence of *Salmonella* in poultry and poultry products ranges from 1% to 65.5% [8-13]. Infected live birds harbor and disseminate *Salmonella* to other birds via lateral transmission, mainly through feces, soil, litter, feeds, water, dust and feathers [14]. In addition, vertical or transovarian transmission of *Salmonella* occurs as eggs from colonized hens are used for the production of chicks. Chicks hatched from these eggs might excrete the bacterium, infecting other chicks [14]. As live birds are processed, the existing bacterium is introduced to poultry production system, and each stage of processing is a potential point for the environment for *Salmonella* contamination. *Salmonella* could, therefore, spread from carcass to carcass along the processing stages. In wet markets, the cross-contamination of *Salmonella* is further aggravated, due to poor hygiene and sanitation practices as well as the extensive amount of manual handling. Subsequently, *Salmonella* survives on chicken carcasses and other processed chicken products.

**Figure-1:**
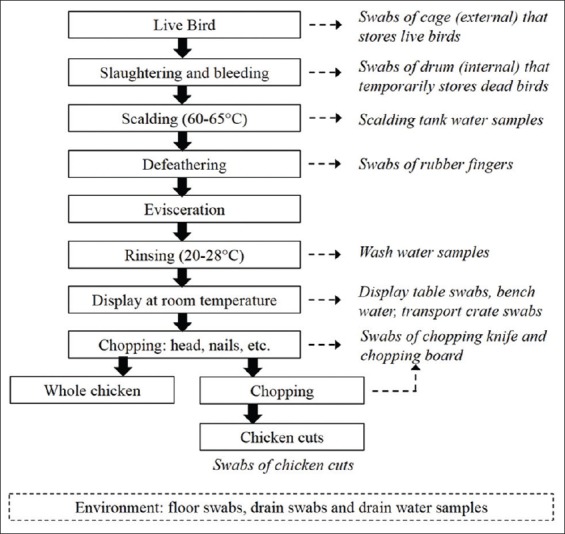
Flow diagram of poultry processing in wet markets with detailed description of *Salmonella* sampling points.

Understanding the contamination sources of *Salmonella* during slaughter and processing are imperative in reducing the prevalence of *Salmonella* in poultry. Numerous studies on the prevalence of *Salmonella* in poultry in Malaysia have been reported [15-17], but these studies focus on the occurrence of *Salmonella* rather than studying the source and dissemination mode of *Salmonella*. There is no information available to indicate whether the poultry is contaminated during processing in wet markets and small processing plants or during retailing, although studies have shown that live poultry is contaminated at the farm. *Salmonella* is present in the intestines, feathers, feet, and poor cleaning and sanitation will lead to cross-contamination and colonization of various sites in the wet markets and plants. The presence of high moisture in the environment of wet markets and poultry processing plants are ideal for the establishment and colonization by foodborne pathogens. Chmielewski and Frank [18] in an extensive review on biofilms and citing studies by other researchers observed that improperly cleaned surfaces promote biological soil build-up, and, in the presence of water, contribute to the development of bacterial biofilms which may contain pathogenic microorganisms. They also observed that *Salmonella* can easily attach and form biofilms on surfaces found in food processing plants, including plastic, cement, and stainless steel. According to our observation, poultry processing in wet markets and small scale processing plants in Malaysia involves constant rinsing steps as shown in Figure-1. Wet environment encountered in poultry processing plants is ideal for biofilm formation. Studies have shown that *Salmonella* prevalent in poultry processing environment can be isolated from poultry processing equipment, especially in the slaughter and evisceration areas [19-21].

Arumugaswamy *et al*. [22] observed that 39%, 35% and 44% of chicken parts, livers and gizzards were contaminated with *Salmonella*. Rusul *et al*. [15] reported that in Malaysia, the incidence of *Salmonella* in poultry carcasses obtained from wet markets and poultry processing plants ranged from 38.3% to 50%. In a recent study, using the most probable number method in combination with multiplex polymerase chain reaction, Thung *et al*. [23] reported that the occurrence of *Salmonella* spp., *Salmonella* Enteritidis, and *Salmonella* Typhimurium in 120 chicken meat samples obtained from wet markets and hypermarkets was 20.80%, 6.70%, and 2.50%, respectively. Therefore, this study was undertaken to provide a detailed insight on the prevalence of *Salmonella* serotypes at different stages of poultry processing. The samples examined consisted of whole chicken carcasses, chicken cuts, and carcass contact surfaces such as utensils and processing equipment as well as environmental samples collected from wet markets and small scale processing plant located in Penang and Perlis.

## Materials and Methods

### Ethical approval

Live animals were not used in this study, hence ethical approval was not necessary. Poultry and environmental samples were collected from local wet markets and small scale processing plant.

### Sampling

A total of 182 poultry (30/182) and environmental samples (152/182) were collected at random on separate occasions from wet markets and small scale processing plant, during the period of October 2014 to July 2015 in Penang (108/180) and Perlis (74/180), Malaysia. Figure-1 shows the flow diagram of poultry processing in wet markets with a detailed indication of *Salmonella* sampling points. Environmental and process related samples - such as floor, display table, chopping board, knife, cage, drum, defeathering machine, transport crates, and drain crevices - were swabbed using 3M™ Sponge-Sticks (3M, USA) as per manufacturer’s instructions. The swab-sampling method described by Gill *et al*. [24] was adopted for the whole chicken carcass and chicken cuts. Scalding tank water, wash water (water used for washing carcass after defeathering), bench wash water (stagnant fluids on display table), and drain water samples were collected in sterile Schott bottles (100 ml). All samples were transported in a polystyrene box containing ice and analyzed immediately on reaching the lab.

### Culture-based method

Enumeration and isolation of *Salmonella* in poultry and its related process and environmental samples was carried out according to ISO 6579:2002 Horizontal Method [25], with slight modification. Processing and environmental swab samples (25 g) were pre-enriched in 225 ml of buffered peptone water (BPW) (3M, USA). Water samples (25 ml) were pre-enriched in 225 ml of BPW as well. Both test (swab and water) samples were incubated at 37±1°C for 24±2 h. Upon pre-enrichment, 0.1 ml aliquot of test samples were transferred into 9.9 ml of rappaport vassiliadis soya (RVS) broth and were incubated at 42±1°C (RVS) for 24±2 h. Concurrently, 1 ml aliquot of pre-enriched test samples was transferred into 9 ml Muller-Kauffmann tetrathionate-novobiocin (MKTTn) broth and was incubated at 37±1°C (MKTTn) for 24±2 h. One loop of RVS and MKTTn were streaked onto following selective agars: Xylose lysine deoxycholate agar, rambach agar, xylose lysine tergitol-4 agar, and hektoen enteric agar. The selectice agar plates were then incubated at 37±1°C for 24±2 h. Presumptive *Salmonella* colonies were chosen and purified and subjected to the biochemical tests such as triple sugar iron and lysine iron test. Media used for selective enrichment, selective plating, purification, and biochemical tests were purchased from Merck, Germany. *Salmonella* isolates were also serologically confirmed by using polyvalent O and H antisera (BD Franklin Lakes, USA). The *Salmonella* isolates were serotyped at Public Health Laboratory, Perak, Ministry of Health Malaysia.

## Results

*Salmonella* was isolated from 161 out of 182 samples (88.46%). All chicken carcasses (15/15) and chicken cuts/portions (15/15) were positive for *Salmonella* (Table-1). *Salmonella* was also isolated from all environmental samples (floor, bench wash water, wash water and drain water) and contact surface of equipment (transport crate, cage, drum, knife, chopping board, and display table). *Salmonella* was detected in 91.67% (11/12), 83.33% (5/6), and 66.67% (4/6) defeathering machines, drain, and apron swabs, respectively (Table-1). All (0/17) scalding tank water samples were negative for *Salmonella*. Results in Table-2 show that the incidence of *Salmonella* was very high in wet markets located in Penang (83.33-91.67%) and Perlis (80.00-92.86%). This suggests that *Salmonella* is persistent in the environment of wet markets of both states, indicating colonization and survival in biofilms.

**Table-1:** Detection of *Salmonella* in various samples obtained from wet markets and small scale processing plant located in Penang and Perlis.

Samples tested	Number of samples tested	Number of positive samples	Prevalence (%)
Cage	11	11	100.00
Drum	12	12	100.00
Scalding tank water	17	0	0.00
Defeathering machine	12	11	91.67
Wash water	8	8	100.00
Chopping board	17	17	100.00
Whole chicken	15	15	100.00
Chicken cuts	15	15	100.00
Knife	14	14	100.00
Display table	17	17	100.00
Floor	17	17	100.00
Drain swab	6	5	83.33
Drain water	9	9	100.00
Crates	4	4	100.00
Bench wash water	2	2	100.00
Apron	6	4	66.67
Overall	182	161	88.46

**Table-2:** Incidence of *Salmonella* at different wet markets and small scale processing plant in Penang and Perlis.

Location	Visit	Number of positive sample	Total number of sample	Prevalence (%)
Penang				
Jelutong^a^	1	10	11	90.91
	2	10	11	90.91
	3	10	11	90.91
	4	10	11	90.91
Batu Lancang^a^	1	10	12	83.33
	2	10	12	83.33
	3	11	12	91.67
Lip Sin^a^	1	5	6	83.33
	2	5	6	83.33
	3	5	6	83.33
Tun Sardon^a^	1	9	10	90.00
Grand total		95	108	87.96
Perlis				
Pauh^a^	1	12	14	85.71
	2	13	14	92.86
Mata Ayer^a^	1	12	13	92.31
	2	12	13	92.31
SSPC^b^	1	8	10	80.00
	2	9	10	90.00
Grand total		66	74	89.19

a Wet markets,

b Small scale processing plant

Seventeen different *Salmonella* serovars were isolated from samples obtain from wet markets and small-scale processing plants located in Penang and Perlis. The various *Salmonella* serovars isolated and their distribution is presented in Table-3. The predominant serovars isolated were *Salmonella* Albany (57/161), *Salmonella* Corvallis (42/161), and *Salmonella* Brancaster (37/161). *Salmonella* Enteritidis (5/161), *Salmonella* Typhimurium (4/161), *Salmonella* Florian (3/161), and *Salmonella* Braenderup (3/161) were also detected. The prevalence of other serovars such as *Salmonella* Give, *Salmonella* Weltevreden, *Salmonella* Kivu, *Salmonella* Sarajane, *Salmonella* Haifa, *Salmonella* Indiana, *Salmonella* Kentucky, *Salmonella* Oyonnax, *Salmonella* Chester, and *Salmonella* Stanley was very low (<2). Among the 17 different *Salmonella* serovars, nine different serovars were isolated from wet markets in Penang, and the predominant serovars were *S*. Corvallis (34/95), *S*. Albany (28/95), and *S*. Brancaster (21/95). 14 different *Salmonella* serovars were isolated from wet markets, and small-scale processing plant in Perlis and the most predominant serovars were *S*. Albany (29/66), *S*. Brancaster (16/66), and *S*. Corvallis (8/66).

**Table-3:** *Salmonella* serovars isolated from different wet markets and small scale processing plant located in Penang and Perlis.

*Salmonella* serovars	Penang				Perlis			Number of isolates (% prevalence)
	
	Jelutong	Batu Lancang	Lip Sin	Tun Sardon	Pauh	Mata Ayer	SSPC	
*Salmonella* Albany	13	11	1	3	9	16	4	57 (35.40)
*Salmonella* Corvalis	19	7	6	2	4	1	3	42 (26.09)
*Salmonella* Brancaster	2	13	5	1	10	4	2	37 (22.98)
*Salmonella* Enteritidis	2		2			1		5 (3.11)
*Salmonella* Typhimurium	2		1		1			4 (2.48)
*Salmonella* Florian					1	2		3 (1.86)
*Salmonella* Braenderup				2			1	3 (1.86)
*Salmonella* Give	1							1 (0.62)
*Salmonella* Weltevreden							1	1 (0.62)
*Salmonella* Kivu							1	1 (0.62)
*Salmonella* Sarjane							1	1 (0.62)
*Salmonella* Haifa	1							1 (0.62)
*Salmonella* Indiana				1				1 (0.62)
*Salmonella* Kentucky							1	1 (0.62)
*Salmonella* Oyonnax							1	1 (0.62)
*Salmonella* Chester							1	1 (0.62)
*Salmonella* Stanley							1	1 (0.62)
Overall	40	31	15	9	25	24	17	161 (100.00)

## Discussion

A recent *Salmonella* outbreak in Kedah, Malaysia which resulted in four deaths and 38 cases of hospitalization due to inappropriate storage of raw chicken, followed by insufficient cooking and the subsequent consumption of contaminated chicken dish [26] has underlined the implication of *Salmonella* contamination in poultry. In this study, poultry, poultry contact surfaces of equipment used in poultry processing and environmental samples from wet markets and small scale processing plant were analyzed for the presence of *Salmonella*.

Our study demonstrated a high occurrence (88.46%) of *Salmonella* in poultry, contact surfaces used in poultry processing and environmental samples obtained from wet markets and small scale processing plant. Chicken carcass, chicken cuts/portions, contact surfaces (transport crate, cage, drum, knife, chopping board, and display table), and environmental samples (bench wash water, wash water and drain water and floor) were all positive for *Salmonella*. Arumugaswamy *et al*. [22] reported that the prevalence of *Salmonella* in chicken pieces, livers, and gizzards sampled from retail outlets were 39.4% (13/33), 35.3% (6/17), and 44.4% (8/18), respectively. Similarly, Rusul *et al*. [15] reported 35.5% (158/445) and 50.0% (52/104) broiler carcasses obtained from wet markets and processing plants, respectively, were contaminated with *Salmonella*. However, in a recent study, Modarressi and Thong [16] reported a high prevalence of *Salmonella* (72.7%) in chicken meats sampled during 2006-2009 in Kuala Lumpur. Other researchers [8-13] have reported that the incidence of *Salmonella* to be much lower (1-65.5%) compared to findings of Moderressi and Thong [16]. The major difference between this study and the previous studies could be due to sampling strategy and geographical area. In this study, samples were obtained at the various stages of processing (from slaughter to retailing at the wet markets). In this study, environmental and contact surfaces were also examined to determine the potential for cross-contamination. Thus, the focus of this study was to determine the contamination sites and to trek the dissemination of *Salmonella* during slaughtering and processing. Besides reporting a high prevalence of *Salmonella* in poultry and their processing environments, our results also suggested that frequent contamination of *Salmonella* occurs along the processing line. The previous studies as reported by other researchers only focused on the prevalence of *Salmonella* in chicken carcasses or chicken cuts and chicken by-products such as organs (gizzards, livers, and intestines). This could explain the low prevalence of *Salmonella* compared to our study which focused on the prevalence of *Salmonella* at different stages of processing in wet markets. In our opinion, the high incidence of *Salmonella* in samples obtained from wet markets is due to cross contamination. The prevalence of *Salmonella* in live birds arriving in wet markets might be very low but during processing under unhygienic conditions led to the amplification of contamination of the carcass from various stages along the processing continuum.

The predominant serovars detected in samples obtained from wet markets in Penang and Perlis were *S*. Albany, *S*. Corvallis, and *S*. Brancaster. However, the pattern of predominance was different as *S*. Albany was the predominant serovar in Perlis, followed by *S*. Brancaster and *S*. Corvallis; while in Penang, the predominant serovar was *S*. Corvallis, followed by *S*. Albany and *S*. Brancaster. The serovars isolated in this study were different from those reported by Rusul *et al*. [15]. In their study, Rusul *et al*. [15] isolated 14 serovars from carcass obtained from wet market, with *S*. Muenchen (32.6%), *S*. Enteritidis (19.8%), *S*. Kentucky (17%), and *S*. Blockley (12.8%) as the predominant serovars. In another study, Arumugaswamy *et al*. [22] isolated eight serovars from poultry and giblets, with *S*. Blockley, *S*. Enteritidis, and *S*. Chincol as the most predominant serovars. Two similar studies conducted in Thailand showed that the predominant serovars in Thailand were *S*. Corvallis, *S*. Albany, *S*. Enteritidis, and *S*. Virchow [27], while Chotinun *et al*. [28] reported that *S*. Corvallis, *Salmonella* Rissen, *Salmonella* Hadar, *S*. Enteritidis, *S*. Stanley, and *S*. Weltevreden were the predominant serovars. Bae *et al*. [29], on the other hand, reported *S*. Enteritidis, *Salmonella* Montevideo and *Salmonella* Senftenberg as the most prevalent serovars isolated from poultry slaughterhouses in Korea. In general, the distribution of *Salmonella* serovars varies over time, different geographical locations, production scale and the country’s development status [30]. Type of serovars and their distribution pattern is almost constant in developed countries while it varies greatly with time in developing countries [30]. Some serovars remain dominant over many years, others emerge, re-emerge or decrease over time [31]. Moreover, a rapid international trade in food and agricultural products has eased the interpolation of *Salmonella* serovars across the geographic boundaries of importing countries [32].

It is worth to note that the prevalence of *Salmonella* has considerably increased over the years in Malaysia, suggesting that poultry from wet markets can be an important vehicle for the transmission *Salmonella* spp. This may be attributed to increased poultry production and the stress induced during poultry transportation [22], while in wet markets, the high prevalence of *Salmonella* can be attributed to poor hygiene and sanitation practices. The incidence of *Salmonella* in poultry at the farm level might be low but during transportation in overcrowded cages over a long distance will lead to stress among the poultry. It is a well-known fact there is an increase in shedding of *Salmonella* or other enteric pathogens if live poultry or life animals are subject to stress especially during transportation [33,34]. Heyndrickx *et al*. [34] in an epidemiological study on the dissemination of *Salmonella* from hatchery to slaughterhouse observed a significant increase in *Salmonella* contamination during transport. Poultry birds can be colonized by *Salmonella* via vertical and lateral transmission (environment) route [14]. The live birds are loaded and transported to wet market in plastic cages, and during transportation period, the cages may get contaminated by feces, litters and feathers. In this study, all the cage samples were positive for *Salmonella*, indicating that the cage used to transport live birds can be a source of contamination. The impact of unclean and recycled transport crate for live bird transportation have been well documented [35].

Many studies have shown that cross-contamination of poultry can occur during processing. In the processing plants and wet markets, the sources of contamination can be workers, equipment such as defeathering machines, scalding water and tanks, chilling tanks, floors, drains work benches, and knives. *Salmonella* serovars can also survive for a long time in biofilms which are formed on surfaces, and these biofilms tend to protect *Salmonella* from detergents and sanitizers. In wet markets, the live birds are killed, scalded in hot water, plucked and eviscerated, mostly by hand. Extensive human handling involved, at each stage of processing, contributes to cross-contamination. In this study, it was found that most carcass contact and environmental samples collected along chicken processing line were consistently contaminated with *Salmonella*. All scalding tank samples were negative for *Salmonella*. The scalding process involves immersing the carcass in hot water at 50-58°C for 4 min, to loosen the feathers from skin pores, which facilitates the defeathering process [36]. Due to the high temperature employed during scalding, *Salmonella* was not isolated from the scalding water samples. After scalding, the carcasses are subjected to defeathering and evisceration. Both processes are of major concerns in poultry industry as it may lead to cross contamination and recontamination of carcasses [37]. The rubber fingers in defeathering machine harbor pathogens, which in turn contaminates the carcass while careless harvesting of entrails and intestines manually could contaminate the carcass during evisceration as a result of intestinal content spillage [37]. After defeathering, the carcasses are rinsed with water. In this study, all the wash water samples were positive for *Salmonella*. In wet markets, where *Salmonella* was isolated from wash water, suggests limited water supply and the water used for rinsing of carcass is recycled frequently. The presence of *Salmonella* on chopping board, knife and display table suggest this pathogen have colonized these contact surfaces through formation of biofilms and thus able to survive cleaning. We observed there are no systematic cleaning or sanitation programs in the wet markets. The cleaning is carried out by hosing of loose soils such as dirt, blood, feather and tissue residues. We also observed potholes, puddles of water and the drain walls are covered with algae and due to poor gradient water flow is slow.

## Conclusions

This study describes the prevalence of *Salmonella* contamination in the whole chicken carcass, cut chickens, processing and environmental samples obtained from wet market and small-scale processing plant in Perlis and Penang, Malaysia. The study found contamination of *Salmonella* in whole and cut chickens, utensils and equipment used in processing, in wash, scalding tank, bench and drain water and floor around the wet markets and small scale processing plant, indicating potential sources and routes of the *Salmonella* transmission in poultry, as well as its processing environment. Predominant *Salmonella* serotypes isolated were *S*. Albany, *S*. Corvallis and *S*. Brancaster. The relatively high prevalence in chickens and its processing environment in local wet markets and small scale processing plant indicates that poultry is undoubtedly a major potential source of human salmonellosis. These results call for urgent attention as such prevalence are imminent risk to public health. This research also highlights the need of implementing a stricter hygiene and sanitation standard in local wet markets and small scale processing plant to reduce the incidence of *Salmonella*. As poultry processing involved multiple stages, there is evidence that some of the processing steps facilitate the contamination of poultry carcasses.

## Authors’ Contributions

GR conceived and designed the study. HNidaullah and NA carried out sampling and laboratory analysis. AKSS, LOC and HNurul analyzed and interpreted the data. TPT and FWZA assisted with the serovar typing. All authors wrote, revised and approved the manuscript.
